# A preliminary investigation of short-term cytokine  expression in gingival crevicular fluid secondary to high-level orthodontic forces and the associated root resorption: case series analytical study

**DOI:** 10.1186/s40510-017-0177-x

**Published:** 2017-08-07

**Authors:** Rajiv Ahuja, Moahmmed Almuzian, Alamgir Khan, Dana Pascovici, Oyku Dalci, M. Ali Darendeliler

**Affiliations:** 1 Discipline of Orthodontics, Faculty of Dentistry, University of Sydney, Sydney, Australia; 2 Oxford University Hospitals NHS Foundation Trust, Oxford, UK; 3 Eastman Dental Hospital, UCLH NHS Foundation Trust, London, UK; 4 Australian Proteome Analysis Facility, Macquarie University, Sydney, Australia

## Abstract

**Background:**

Orthodontically induced iatrogenic root resorption (OIIRR) is an unavoidable inflammatory process. Several factors claimed to be related to the severity of OIIRR. Orthodontic forces cause micro-trauma to the periodontal ligament and activate a cascade of cellular events associated with local periodontal inflammation. The purpose of this split-mouth study were (1) to investigate the changes in cytokine profile in the gingival crevicular fluid (GCF) secondary to heavy orthodontic forces and (2) to compare the cytokine expression between participants showing high and low root resorption.

**Methods:**

Eight participants requiring maxillary first premolar extractions involved in this study. The teeth on the tested side (TS) received 225 g of controlled buccal tipping force for 28 days, while the contralateral teeth act as a control (CS). GCF was collected from both TS and CS teeth at 0 h (prior to application of force) and 3 h, 1 day, 3 days, 7 days and 28 days after the application of force, and analysed with multiplex bead immunoassay to determine the cytokine levels.

**Results:**

Statistically significant temporal increase was found in the TS teeth for tumour necrosis factor alpha (TNF-α) at 3 h and 28 days (*p* = 0.01). Interleukin 7 (IL-7) significantly peaked at the 28th day. Comparing cytokine profile for participants with high and low root resorption (>0.35 and <0.15 mm^3^, respectively), the levels of GM-CSF was significantly greater in low root resorption cases (*p* < 0.05). The amounts of root resorption which craters on mesial, distal surfaces and middle third region were significant in the TS teeth (*p* < 0.05).

**Conclusions:**

IL-7 and TNF-α (pro-resorptive cytokine) increased significantly secondary to a high-level of orthodontic force application. Significantly high levels of granulocyte macrophage colony-stimulating factor (anti-resorptive cytokine) were detected in mild root resorption cases secondary to high-level orthodontic force application. A future long-term randomised clinical trial with larger sample taking in consideration gender, age and growth pattern distribution would be recommended.

## Background

Orthodontically induced iatrogenic root resorption (OIIRR) is an unavoidable inflammatory process that results in a loss of substance from mineralized cementum during orthodontic tooth movement, and it occurs when resorption outpaces healing [1]. OIIRR ranges from mild (0–2 mm) to severe form (more than 4 mm) [2]. Mild OIIRR is common in almost 90% of orthodontic participants [3], while, luckily, the severe form develops in approximately 4% of participants and more frequent in adults than in adolescents [4].

Several factors claimed to be related to the severity of OIIRR such as race, gender, age and genetic and local factors such as habits, traumatised teeth, pre-existing root resorption, hypo-functional periodontium, duration of treatment and magnitude and direction of force [5, 6]. A systematic review by Weltman et al. showed insufficient conclusive findings in the clinical management of root resorption, still, there is evidence to support the use of light forces, especially with incisor intrusion [3].

Orthodontic forces cause micro-trauma to the periodontal ligament and activate a cascade of cellular events associated with local periodontal inflammation [7]. The upregulation of cytokines such as interleukin (IL)-1 beta (IL-1β), IL-8, tumour necrosis factor alpha (TNF-α) and prostaglandin E2 (PGE_2_) following orthodontic force application has been observed in many studies [8–12].

Cytokines were sub-classified into two main groups: pro-resorptive and anti-resorptive. Pro-resorptive cytokines such as interleukin-1 superfamily (i.e. IL-1β), IL-6, IL-7, IL-8 and TNF, TNF-α and IL-1β directly induce osteoclastogenesis and promote osteoclast function [13–16]. Similarly, IL-6 acts synergistically, with IL-1 and TNF-α, on osteoclastogenesis to promote osteoclast function [13–16]. IL-7 works indirectly through the induction of TNF-α, an important augmenter of receptor activator of nuclear factor kappa-B ligand (RANKL)-mediated osteoclastogenesis [17], while IL-8 enhances RANKL expression [18]; both increase osteoclast generation and activate osteoclasts. On the other hand, an anti-resorptive cytokines such as IL-4 and interferon gamma (IFN-γ) suppress osteoclastogenesis and T cell of RANKL-induced osteoclastogenesis respectively [19, 20]. Granulocyte macrophage colony-stimulating factor (GM-CSF) is another anti-resorptive cytokine that inhibits bone resorption along with IL-4, IL-10, IL-13, IL-18 and IFN-γ [21]. Cementoclasts have similar precursor cells [22] and enzymatic and metabolic properties to an osteoclast [23] and share the common regulatory mechanism of cellular resorption of mineralized tissues such as bone and dentine [24]. The differentiation and activation of pre-cementoblasts under both physiologic and pathologic OIIRR conditions require the expression of RANKL, osteoclastogenesis inhibitory factor (OPG) and macrophage colony-stimulating factor (M-CSF) by the dental cells [25].

Cellular and tissue reactions start in the initial phase of tooth movement, immediately after force application. The rate of connective tissue turnover during tooth movement is dependent on the individual difference in anatomic structures, mineral density, the level of cytokines and variations in metabolic capacity [26]. This variability in the levels of pro-resorptive and anti-resorptive cytokines in the micro-environment (cytokines’ profile) of periodontal ligament (PDL) may also lead to a difference of OIIRR in response to a similar amount of orthodontic force. The use of gingival crevicular fluid allows a non-invasive means of detecting OIIRR during tooth movements.

The aims of this study were to investigate the change in the cytokine profile in gingival crevicular fluid (GCF), during the initial and lag phases of tooth movement after applying heavy orthodontic forces, and to compare the cytokine expression between participants showing the high and low volume of root resorption. The null hypothesis is that there is no change in the cytokine expression and no correlation with the degree of OIIRR secondary to orthodontic force application.

## Methods

### Sample

Ethical approval was obtained from South Western Sydney Local Health Network Ethics Review Committee (protocol no. X11-0028 and HREC/11/RPAH/37). The study sample consisted of eight consecutive participants (6 males, 2 females; mean age = 16.4 years; range 13.9–22.9).

All participants required orthodontic treatment with extraction of the maxillary first premolars and met clearly defined inclusion criteria including (1) class I malocclusion, (2) class I skeletal base, (3) average vertical height, (4) absence of obvious facial asymmetry, (5) normal growth and development of the dentition and (6) radiographical signs of complete apexogenesis of the upper first premolars. All participants with a history of medical problem(s) that related to abnormal dental development, history of trauma or bruxism and caries or dental or orthodontic treatment were excluded. Clinical periodontal examinations including periodontal probing pocket depth, bleeding on probing and attachment level of the upper first premolars was undertaken before and during treatment to exclude any periodontal disease. Informed consents from all subjects were obtained at the first visit.

### Study design

**Fig. 1:**
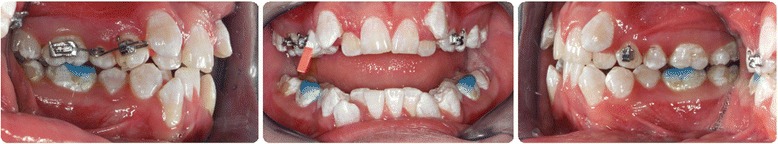
Intraoral views, appliance design and GCF collection

A split-mouth study design with the similar experimental setup described by Srivicharnkul et al. was adopted [27]. SPEED orthodontic brackets (Strite Industries Cambridge, Ontario, Canada), 0.022″ × 0.028″ slot size, were bonded to the upper first permanent premolars and the upper first permanent molar. A 0.017 × 0.025-inch TMA (Beta III titanium, 3M Unitek, Monrovia, California) cantilever spring was used to apply 225 g of buccally directed force to the first premolars in the test side (TS) for 28 days. The orthodontic force was measured to the nearest gram with a strain gauge (Dentaurum, Ispringen, Germany). To disocclude the occlusion and prevent occlusal forces, light cured glass ionomer cement (Transbond; 3M Unitek, Monrovia, Calif) was bonded bilaterally onto the occlusal surfaces of the lower first molars (Fig. 1).

### Gingival crevicular fluid collection and determination of cytokine levels

After isolating the teeth, GCF was collected from the mesiobuccal aspect of both TS and control side (CS) teeth at the following time periods: 0 h (prior to application of force), 3 h, 1 day, 3 days, 7 days and 28 days after the application of force.

The protocol for GCF collections involved gentle removal of visible plaque at both sites followed by washing and drying the premolar regions using air syringe to avoid contaminating the paper strip (Periopaper, Harco, Tustin, CA, USA) by the plaque. Then, paper strips were inserted for 1 mm into the gingival crevice and left there for 30 seconds. Periotron 8000 (Periotron 8000, Oraflow Inc., New York, USA) was used to measure the volume of GCF in each paper strip. Microcentrifugee tubes containing paper strips were thawed, and GCF was eluted from the paper strips using 50 μL of phosphate-buffered saline (pH 7.2) containing protease inhibitor cocktails (Sigma, St Louis, USA) and 0.05% (*v*/*v*) Tween 20 (Sigma, St Louis, USA). Following micro-centrifuging, the paper strips were immediately frozen at −80 °C for further immunoassay analysis to determine cytokine levels [28]. Multiplex bead immunoassay (product no. HSCYTMAG-60SK EMD Millipore corporation) was used to determine the levels of IL-1β, 2, 4, 5, 6, 7, 8, 10, 12 and 13, GM-CSF, INF-γ and TNF-α simultaneously using Bio-Plex 200 systems (Bio-Rad, CA, USA).

### Processing of the extracted teeth

At the end of day 28, the TS and CS teeth were extracted atraumatically to avoid damage to the root cementum. The teeth were processed by immersing extracted them in a solution of sterilised deionised water (Milli-Q; Millipore, Bedford, Mass). Then, the teeth were treated for 10 min in an ultrasonic bath to remove residual PDL and soft tissue fragments [29]. The remaining visible PDL was manually cleaned using a damp gauze swab before final disinfection using 70% alcohol for 30 min. Finally, the disinfected teeth were stored in Milli-Q at room temperature (23 ± 1 °C) with 50% ± 10% relative humidity before scanning.

### Micro-CT scanning procedure

The extracted teeth were scanned individually for 60 minutes per tooth using SkyScan 1172 desktop X-ray micro-tomograph system (SkyScan, Aartselaar, Belgium) which allows 180° of rotation. Scanning extended from the root apex to 2 millimetres below the cementoenamel junction (CEJ) with a rotation step of 0.45° and an exposure time of 1.904 seconds. This scanning procedure produced a total of 420 X-ray images per tooth, which were stored as 16 bit TIFF (Tagged Image File Format) files before being reconstructed.

Reconstruction was performed using SkyScan’s volumetric reconstruction software (NRecon, version 1.6.8, Aartselaar, Belgium) [30]. An ImageJ variant (FIJI) was used to isolate and export individual lacunae from axial slices. An ImageJ macro (ACMM), the University of Sydney (Enigma), was utilised to measure the volume of each root lacuna uniformly. The lacunae were separated according to their location in the coronal, sagittal and axial plane. One operator carried out volumetric measurements of OIIRR twice at an interval of 2 weeks in order to measure intra-operator variability.

### Statistical analysis

To normalise the raw cytokine data, the concentration of each cytokine was considered. An analysis of variance was undertaken separately for each cytokine to check for overall changes with time. ANOVA *p* values were adjusted for multiple testing using the Benjamini and Hochberg FDR correction.

The non-parametric alternative to ANOVA (Kruskal-Wallis test) was also run side by side for reference, using the raw data to evaluate the significance of the difference between the groups and within each time point. Cytokine changes between particular time points were calculated together by using Tukey’s Honest Significant Differences as implemented in Tukey’s HSD function of the *R* statistical package. In a similar fashion, a one-way ANOVA was run to ascertain cytokine changes over time only.

## Results

### Immunoassay results

Intra-operator reliability showed that cytokines IL-1β, IL-4, IL-6, IL-7, IL-8, IFN-γ, GM-CSF and TNF-α were detected reliably by the immunoassay (*p* > 0.05), while IL-2, IL-5, IL-10, IL-12 and IL-13 were not reliably detected as they were missing in more than 50% (Table 1).

**Table 1:** Summary of *p* values from ANOVA analyses with time and treatment factors using the test and control teeth

Cytokine	Time *p* value	BH corrected Time *p* value	Treatment *p* value	Interaction *p* value	Time Kruskal *p* value	Kruskal BH-adjusted	Number measurements	Missing measurements (%)
IL-1β	0.27	0.55	0.15	0.80	0.24	0.56	90	6.25%
IL-2	0.22	0.52	0.26	0.23	0.35	0.65	29	*69.79%*
IL-4	0.66	0.89	0.05	0.83	0.49	0.66	71	26.04%
IL-5	NA	NA	NA	NA	0.26	0.56	4	*95.83%*
IL-6	0.60	0.89	0.65	0.95	0.59	0.70	76	20.83%
IL-7	0.05	0.24	0.89	0.89	0.03	0.18	52	45.83%
IL-8	0.88	0.96	0.64	0.76	0.92	0.94	96	0.00%
IL-10	0.80	0.96	0.85	0.74	0.44	0.66	14	*85.42%*
IL-12p.70	0.16	0.47	0.12	0.26	0.15	0.48	38	*60.42%*
IL-13	0.06	0.24	0.44	0.62	0.09	0.39	22	*77.08%*
IFN-γ	0.99	0.99	0.42	0.75	0.94	0.94	87	9.38%
GM-CSF	0.47	0.81	0.17	0.68	0.51	0.66	95	1.04%
TNF-α	0.01	0.08	0.95	0.94	0.00	0.05	95	1.04%

Cytokines with more than 50% missing observations are presented in italics

**Fig. 2:**
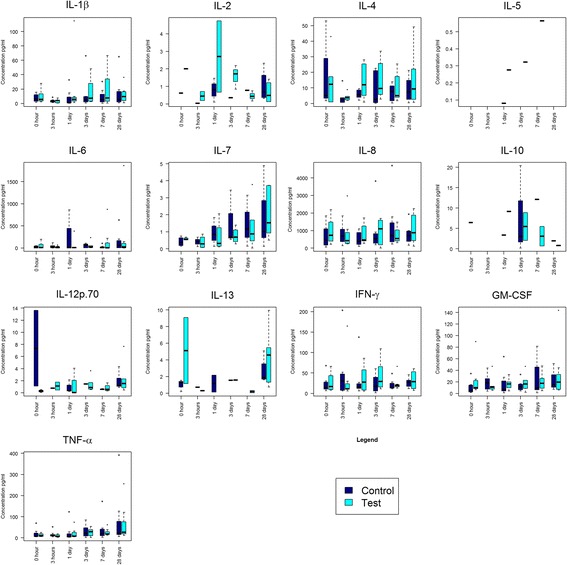
Box plot distribution of raw data for GCF cytokines by time and treatment, separately for each cytokine. The *horizontal line* within the box plot represents the median, vertical line from the *boxes* (*whiskers*) indicating variability outside the upper and lower quartiles; squares and circular points represent the observation point (outliner)

Figure 2 illustrates the overall look of the raw cytokine data with time and treatment factors. Upward time-dependent changes were detected for IL-7 and TNF-α, both by ANOVA and the non-parametric version (Kruskal-Wallis test), while treatment-dependent changes were detected for IL-4. Additionally, the analysis of variance identified the cytokine that showed overall temporal differences without identifying precisely which pairs of points exhibit a significant difference (Table 1). The results in Table 2 summarise the time points that show significant differences in overall cytokine abundance. The increase in cytokine abundance after day 28 (IL-7 increased at day 28 compared to that at hour 3 ; TNF-α increased at day 28 compared to that at hour 0, 3 and 24) confirms the visual pattern of the increase in the data. When the same post hoc analysis was repeated for the TS teeth, the time differences for TNF-α were found to be significant. This finding is consistent with a broad trend of noticeable increase at day 28 (Fig. 2).

**Table 2:** Significant temporal differences as based on post hoc analysis of ANOVA results using Tukey’s Honest Significant Differences

Cytokine	Time points	Difference in means	*p* value after adjustment for multiple comparisons
IL-7	28 days–3 h	1.56	0.02
TNF-α	28 days–0 h	1.05	0.04
TNF-α	28 days–3 h	1.23	0.01
TNF-α	28 days–1 day	1.13	0.02
Significant temporal differences for TS teeth only			
TNF-α	28 days–3 h	1.53	0.01

### Cytokine comparison in high and low OIIRR cases

Of the eight participants enrolled in this study, (i) three participants were identified as having high OIIRR (>0.35 mm^3^), (ii) three showed low OIIRR (<0.15 mm^3^) and (iii) two participants presented with OIIRR between 0.15 and 0.35 mm^3^, who were excluded from the analysis. This categorisation was based on the mean difference of total volume of OIIRR between the TS and CS teeth.

**Fig. 3:**
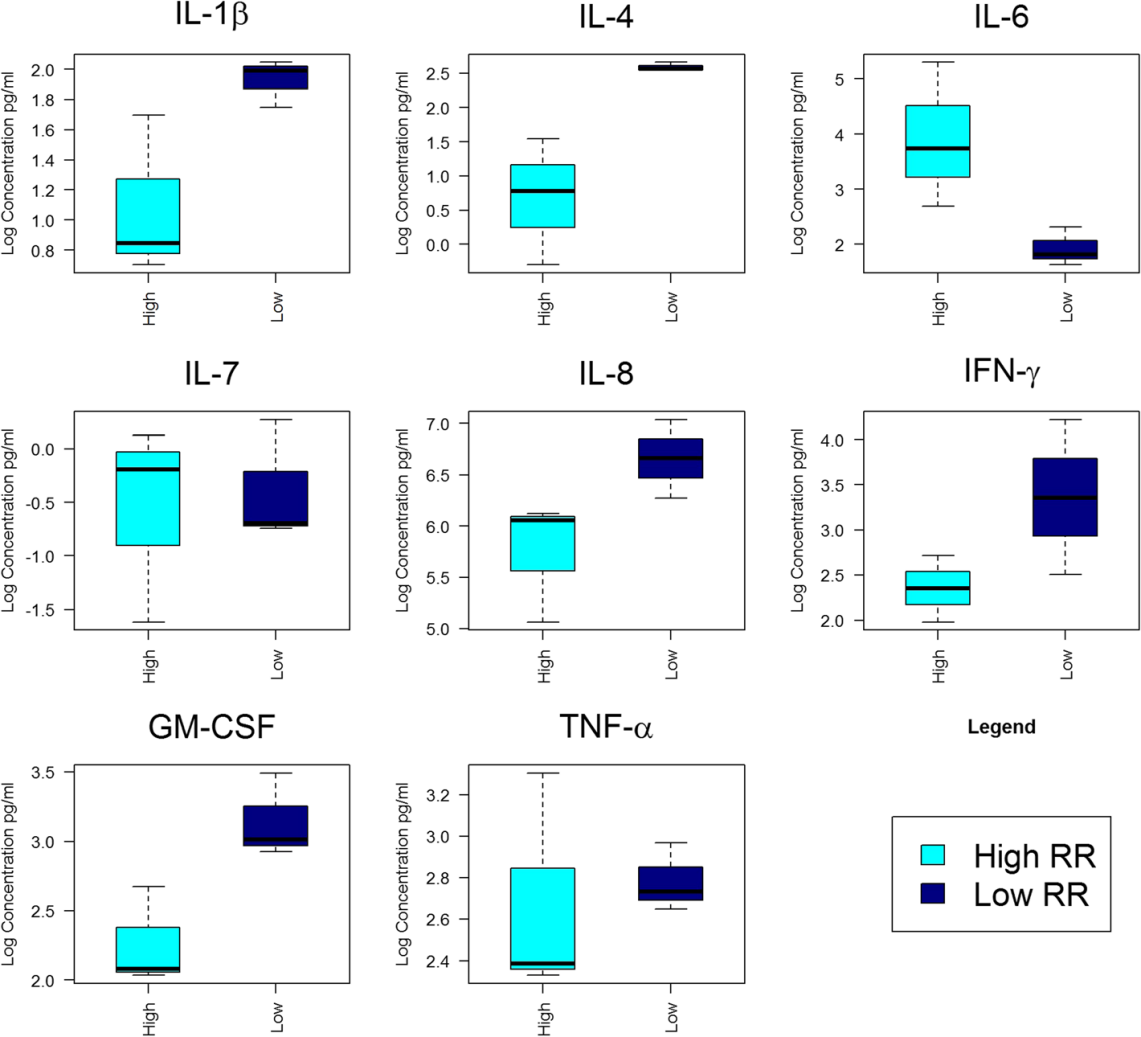
Box plot distribution for average cytokine values from high and low root resorption participants

A *t* test for cytokines that only detected in more than 50% of the participants (i.e. IL-1β, IL-4, IL-6, IL-7, IL-8, IFN-γ, GM-CSF and TNF-α) were undertaken. Average values of those individual cytokines were generated separately for participants with high and low OIIRR (Fig. 3). Though the level of anti-resorptive cytokine GM-CSF level was significantly higher in low OIIRR cases (*p* = 0.03), other cytokines showed no differences (*p* > 0.05).

### Micro-CT assessments of OIIRR at different root surface and positions

Table 3 shows the OIIRR at different surfaces and vertical position. It is obvious that total OIIRR in TS is significantly higher than in CS, in particular, at the middle third of the roots and at both mesial and distal surface of the roots (*p* < 0.05).

**Table 3:** Root resorption means and paired *t* test *p* values for test and control sites, total, by vertical thirds and by surface

	Control side mean	Test side mean	Differences mean	Paired *p* values
Total	0.038	0.292	0.254	0.005
Cervical	0.020	0.046	0.026	0.33
Middle	0.000	0.153	0.153	0.02
Apical	0.027	0.066	0.039	0.11
Buccal	0.003	0.020	0.017	0.11
Palatal	0.002	0.018	0.016	0.3
Mesial	0.025	0.115	0.091	0.02
Distal	0.008	0.138	0.130	0.01

## Discussion

The null hypothesis of the study, stating that there is no change in the cytokine expression and no correlation with the degree of OIIRR secondary to orthodontic force application, was rejected.

### Pro-resorptive cytokines

In the present study, the concentration of IL-1β in the TS teeth increased initially, peaked at days 1 and 7 and then decreased. This result indicates that IL-1β is expressed in the GCF during orthodontic tooth movement similar to that in the previous findings [31, 32]. Though no statistically significant differences were noted in IL-1β levels between the TS and CS teeth at any time points, this indicates that the increase in IL-1β level might be associated with inflammatory response secondary to orthodontic mechanical stress [33].

Similarly, the change in the IL-6 level was statistically insignificant in agreement with Basaran and colleagues [34]. Ren et al. [11] reported an opposite finding; they observed an increase in levels of IL-6 at early hours of tooth movement which peaked after hour 24. Moreover, the concentration of IL-8 in TS fluctuated during the experimental phase, but there was no considerable change between the TS and CS. This finding is parallel to the outcomes of Tuncer et al. [8] but contradicting with those by Basaran et al. [34] and Ren et al. [11]. In fact, the latter two studies reported a significant decrease in the IL-8 level at days 7 and 30 of force application respectively. These dissimilarities, in the detected levels of IL-6 and IL-8, in comparison to other studies may be due to the variation in the used appliance systems, force levels, small sample size and/or some individual variations.

On the other hand, there was a statistically significant difference (*p* = 0.01) in the level of IL-7 and gingival TNF-α between TS and CS that peaked after day 28, similar to that in another study [35]. The increase in the level of TNF-α at day 28 indicates a presence of localised inflammation secondary to force application; this correlates with the levels of IL-6 and IL-7 that also peaked at day 28.

The boost in the pro-resorptive cytokines signifies their crucial roles in stress-induced inflammation through stimulating precursor scavenger cells that required for the removal of hyalinized tissue [33]. Also, the increase in the levels of pro-resorptive cytokines, IL-6, 7 and TNF α, might indicate a continuous periodontal remodelling during the lag phase of tooth movement and a cellular prohibiting mechanism.

### Anti-resorptive cytokines

The time-dependent change of IL-4 concentration followed that of IL-1β, i.e. peaked during the period from day 1 to day 3 but then declined. There were also no significant differences between the TS and CS teeth concerning IL-4 concentration. Similarly, the change in the level of IFN-γ among the TS and CS teeth was insignificant. IFN-γ concentration in the TS teeth fluctuated, increasing to its peak at 72 h. The increase in the levels of IL-4 and IFN-γ closely follows that in IL-1β. This unique style of cytokine expression is the result of combined active periodontal remodelling during initial stages of tooth movement and the cellular prohibiting mechanism that prevents additional differentiation and activation of osteoclastic cells.

With regard to GM-CSF level secondary to orthodontic force application, Ren and colleagues identified a significant elevation of anti-resorptive cytokine GM-CSF in juveniles after application of orthodontic force [36]. However, in the present study, the amounts of GM-CSF for the TS teeth dropped immediately after the start of the experimental phase, increased at day 7 and then reached its peak at day 28, but there was no significant difference between the TS and CS teeth at different time scale.

### Cytokines in high and low OIIRR group

Comparing cytokine profile for participants with high OIIRR (>0.35 mm^3^) and those with low OIIRR (<0.15 mm^3^), GM-CSF was increased in low OIIRR cases (*p* = 0.03), while other cytokines showed no significant differences (*p* > 0.05). These results confirm the link between the high levels of anti-resorptive cytokines such as GM-CSF and the reduced root osteoclastic differentiation.

### Micro-CT assessments of OIIRR at different root surface and positions

In this study, the amounts of OIIRR lacunae on the lingual and buccal surface and at apical and cervical third were statistically insignificant; however, it was significant at the pure tension site (mesial and distal surfaces and middle third region) in the TS teeth. These findings are similar to some of the previous studies [37, 38] but contradicting with many others [39–42]. It will be expected that the amount of OIIRR at the compression sites (bucco-cervical and palate-apical regions) would be higher as in the other study [39], however, this was not confirmed in the current study. The differences may be attributed to two reasons. The first reason is called masking effect which is due to the fact that the measurement of the OIIRR in our study was not surface-region-specific, i.e. the whole surface (mesial, distal, buccal or palatal) and the whole radicular regions (cervical, middle or apical third) were assessed. As each of the root regions and surfaces included an overlapped compression and tension site, theoretically, the metabolic changes between the compression and tension sides masked each other and were not distinguishable. Secondly, the differences in the adopted appliance systems, force levels, small sample size and/or some individual variations may explain these dissimilarities.

### Clinical implications, limitations and future considerations

In this study, two of the pro-resorptive cytokines, namely, IL-7 and TNF-α, increased significantly in the TS teeth secondary to heavy force application. This trend might represent the crucial roles of these cytokines in active OIIRR following heavy orthodontic forces. Moreover, as the teeth with low OIIRR demonstrated significantly high level of GM-CSF (anti-resorptive cytokines) secondary to heavy force application, gingival GM-CSF measurement used in our project might be considered in the future as a non-invasive and useful biomarker to identify participants’ susceptibility to severe root resorption.

However, the findings of this study should be taken as an intriguing hypothesis, not as evidence for some reasons. Firstly, the sensitivity of cytokine detections had shown a wide range of variability (approximately ranged from 1 to 96%). Secondly, the lack of method error analysis in our small sample size study indicates that GCF sampling and quantifications of biomarkers are subjected to comparably significant errors [43]. Furthermore, it should be acknowledged that there were some gender imbalance and age heterogeneity in our study which could be considered as one of the main confounders. While it would be ideal to assess the correlation between cytokine expression and OIIRR on various parts of the tooth root, the gingival sulcus is a pool for CGF, and it would be impossible to identify cytokine expression on each surface.

Based on the findings of this study, authors recommend a long-term future randomised clinical trial with a large sample size taking in consideration general factors such as gender, age and growth pattern distribution as well as local factors such as habits, traumatised teeth, pre-existing root resorption and hypo-functional periodontium.

## Conclusions

IL-7 and TNF-α (pro-resorptive cytokine) increased significantly secondary to high-level orthodontic force application. Significantly high levels of GM-CSF (anti-resorptive cytokine) were detected in low OIIRR cases secondary to high-level orthodontic force application. As this study is a preliminary, a future study with larger sample taking in consideration gender, age and growth pattern distribution would be recommended.
